# Atlas-Guided Quantification of White Matter Signal Abnormalities on Term-Equivalent Age MRI in Very Preterm Infants: Findings Predict Language and Cognitive Development at Two Years of Age

**DOI:** 10.1371/journal.pone.0085475

**Published:** 2013-12-31

**Authors:** Lili He, Nehal A. Parikh

**Affiliations:** 1 Center for Perinatal Research, The Research Institute at Nationwide Children's Hospital, Columbus, Ohio, United States of America; 2 Department of Pediatrics, The Ohio State University College of Medicine, Columbus, Ohio, United States of America; Banner Alzheimer's Institute, United States of America

## Abstract

The developmental significance of the frequently encountered white matter signal abnormality (WMSA) findings on MRI around term-equivalent age (TEA) in very preterm infants, remains in question. The use of conventional qualitative analysis methods is subjective, lacks sufficient reliability for producing accurate and reproducible WMSA diagnosis, and possibly contributes to suboptimal neurodevelopmental outcome prediction. The advantages of quantitative over qualitative diagnostic approaches have been widely acknowledged and demonstrated. The purpose of this study is to objectively and accurately quantify WMSA on TEA T2-weighted MRI in very preterm infants and to assess whether such quantifications predict 2-year language and cognitive developmental outcomes. To this end, we constructed a probabilistic brain atlas, exclusively for very preterm infants to embed tissue distributions (i.e. to encode shapes, locations and geometrical proportion of anatomical structures). Guided with this atlas, we then developed a fully automated method for WMSA detection and quantification using T2-weighted images. Computer simulations and experiments using *in vivo* very preterm data showed very high detection accuracy. WMSA volume, particularly in the centrum semiovale, on TEA MRI was a significant predictor of standardized language and cognitive scores at 2 years of age. Independent validation of our automated WMSA detection algorithm and school age follow-up are important next steps.

## Introduction

Very preterm infants are at high risk for neurodevelopmental impairments. By school age, 30-50% of them exhibit cognitive impairment [1,2]. Many preterm infants develop functional deficits even in the absence of visible brain injury on cranial ultrasound. While brain MRI is more sensitive than cranial ultrasound, the full significance of certain imaging findings remains in question. In particular, the significance of white matter signal abnormality (WMSA) on conventional T2-weighted MRI at around term-equivalent age (TEA) [3] as a predictor of later cognitive impairment remains unclear [4]. 

A few studies with neurodevelopmental follow-up have observed a significant negative association between WMSA and developmental quotient at 18 months corrected age [5,6] and intelligence quotient at 9 years of age [7]. Kidokoro et al. [4] also found a significant association with cognitive scores but only in preterm infants with WMSA severe enough to render the periventricular crossroads regions invisible. Conversely, others have not observed an association with any impairment [8-11]. Considering its high incidence of up to 75% [3], WMSA may represent a prematurity-related developmental phenomenon. Multiple crossing fibers and a high content of hydrophilic extracellular matrix in anterior and posterior periventricular white matter regions may contribute to the high signal intensity observed on T2-weighted MRI and lower anisotropy on diffusion MRI [12,13]. 

The limited progress in resolving the above question can partly be attributed to the use of conventional qualitative MRI readings. Such an approach remains subjective and lacks sufficient reliability for accurate and reproducible WMSA diagnosis [14] and may therefore contribute to suboptimal neurodevelopmental outcome prediction. The advantages of quantitative over qualitative diagnostic approaches have been widely acknowledged and demonstrated [6,15-18]. 

For T2-weighted sequences without fluid attenuation, the signal intensity distribution of WMSA greatly overlaps with that of cerebrospinal fluid (CSF), making the distinction between them difficult [18,19]. To overcome this known difficulty, we propose a probabilistic atlas-guided WMSA detection and quantification method. In the context of this work, we define a probabilistic atlas, as a pairing of anatomy template (an averaged anatomy image from a set of normalized anatomy images in common reference space) and its corresponding tissue probability maps (averaged gray matter (GM), white matter (WM), and CSF segmentations from a set of normalized manual segmentations in common reference space). The atlas that embeds tissue distributions (i.e. encodes shapes, locations and geometrical proportion of anatomical structures) is utilized to guide WMSA detections. 

Atlas-guided analysis methods are usually characterized by how the atlases are generated and how the knowledge regarding atlases can be carried forward to target images. The atlas can be constructed using manual/semiautomatic/automatic segmentations of an individual or a group of individuals [20-27]. Our proposed atlas is constructed using a set of manual parcellations of brains, which are optimal and reproducible. We endeavor to reduce bias toward a specific subject and characterize the variability of a similar age population (extremely low birth weight [ELBW, birth weight ≤1000 g] infants) by utilizing anatomical averaging. We are aware of the differences in performance resulting from different spatial normalization procedures despite use of the same atlas and subject [28]. Large deformation diffeomorphic metric mapping (LDDMM) [29] is well suited to our needs for forward warping an individual subject to an atlas as well as backward warping the atlas to the same subject. Due to the reciprocal nature of the LDDMM, the transformation can be reliably accomplished in either direction [30,31]. 

 In this work, we constructed a probabilistic brain atlas, exclusively for very preterm infants and then guided with this atlas, we proposed a fully automated WMSA detection and quantification method using clinically available T2-weighted images. Computer simulations and experiments on clinical MRI data presented very high detection accuracy. Finally, in a prospective cohort of ELBW infants, we demonstrated a significant correlation between objectively quantified WMSA volumes and language and cognitive developmental scores on the third edition of the Bayley Scales of Infant and Toddler Development at two years of age. 

## Methods

### Ethics Statement

The Children’s Memorial Hermann Hospital (CMHH) and The University of Texas Medical School at Houston joint institutional review board (IRB) approved the study. Written informed consent for each infant was obtained prior to enrollment in the study. The consent was approved by IRB and signed by each subject's parent or guardian prior to enrollment and participation in the study.

### Subjects

The study population was derived from a cohort of 50 ELBW infants without any major congenital anomalies, cared for in the NICU of Children’s Memorial Hermann Hospital from 2007 to 2009. Their mean (standard deviation [SD]) gestational age was 25.5 (1.6) with a range of 23 to 30.1 weeks; birth weight was 750.8 (143.1) grams with a range of 468 to 1000 grams; and the postmenstrual age at MRI was 38.7 (2.3) with a range of 34.1 to 43.9 weeks. Nineteen MRI scans were randomly selected for atlas construction from the cohort study. Their mean (SD) gestational age was 25.8 (1.8) with a range of 23.4 to 30 weeks; birth weight was 724.2 (148.4) grams with a range of 489 to 1000 grams; and post-menstrual age at MRI was 38.1 (2.1) weeks with range of 34.1 to 41.7 weeks. 

### MRI acquisition

All infants were imaged with a 3 T Philips scanner using a dual-echo fast-spin-echo sequence with use of an eight-channel SENSE-compatible phased array receive head coil. The imaging parameters used were: TE1 = 8.75ms; TE2 = 175 ms; TR = 10,000 ms; flip angle = 90°; FOV = 180×180 mm^2^; the imaging matrix = 256×256 mm^2^; slice thickness = 2 mm. In this study, T2-weighted images (corresponds to TE2) were used to construct the atlas and perform the analysis. All infants were transported to the MRI scanner by an experienced neonatal transport nurse after feeding, swaddling and placement of silicone ear plugs (E.A.R. Inc., Boulder, CO) and Natus MiniMuffs (Natus Medical Inc., San Carlos, CA) to facilitate natural sleep and attenuate MRI noise. Sedation was not used for any of the cases. All scans were completed under the supervision of a neonatal research nurse and a neonatologist experienced in neonatal MRI. All MRI scans were read by a neuroradiologist using a standardized reporting tool.

### Very preterm infant probabilistic atlas creation

All 19 ELBW infants randomly chosen for the atlas creation, had varying degrees of WMSA ranging from only in the frontal and/or occipital periventricular white matter up to signal abnormalities covering two-thirds of the cerebral WM. The procedures for atlas creation were similar to the general concept for creating an adult/neonatal group-averaged atlas: 1. Extrameningeal tissues were manually stripped and brain tissues (CSF, WM and GM) were manually parcellated using ANALYZE 8.1 software (Biomedical Imaging Resource, Mayo Clinic, Rochester, MN), as previously described in detail in [19]; 2. T2-weighted images and tissue parcellation maps were then resliced to 1 mm isotropic resolution; 3. Out of all candidates, a representative single-subject image with the brain size/shape fitted to general very preterm infant brains at TEA was selected; 4. Each subject scan was non-linearly normalized to the representative subject using LDDMM to create an averaged anatomy image, which worked as a tentative template for the next step; 5. Each subject was again nonlinearly normalized to the tentative template using LDDMM to create the desired averaged anatomy template; and 6. The resultant nonlinear transformation matrices were applied to the corresponding manual parcellation maps to create the averaged tissue probability maps. All normalization was performed using DiffeoMap (www.mristudio.org, Johns Hopkins University). The atlas is available to interested researchers upon request.

### WMSA regions

When it was first reported in preterm neonates [3], hyperintense WMSA was called diffuse excessive high signal intensity on T2-weighted imaging and defined as “excessive” high signal intensity within the periventricular or subcortical WM that was not limited to the normal distribution of periventricular crossroads (also called layers, anterior caps, and posterior arrowheads). However, the boundaries of these crossroads regions are not well defined. Furthermore, diffusion abnormalities within high signal intensity regions of the centrum semiovale appear to be pathologic and associated with lower developmental quotient [6]. Therefore, in addition to subcortical WM and centrum semiovale, we included anterior and posterior periventricular WM in the regions of our interest. In this work, we will investigate the relationship of outcomes with WMSA regional volumes defined at level of the 1. Entire WM; 2. Periventricular WM – on slices beginning with the first appearance of the frontal horns and ending with the last images of the midbody of the lateral ventricles; and 3. Centrum semiovale – defined as the two axial slices above the last slice of the midbody of the lateral ventricles. 

### Probabilistic atlas-guided WMSA detection

**Figure 1:**
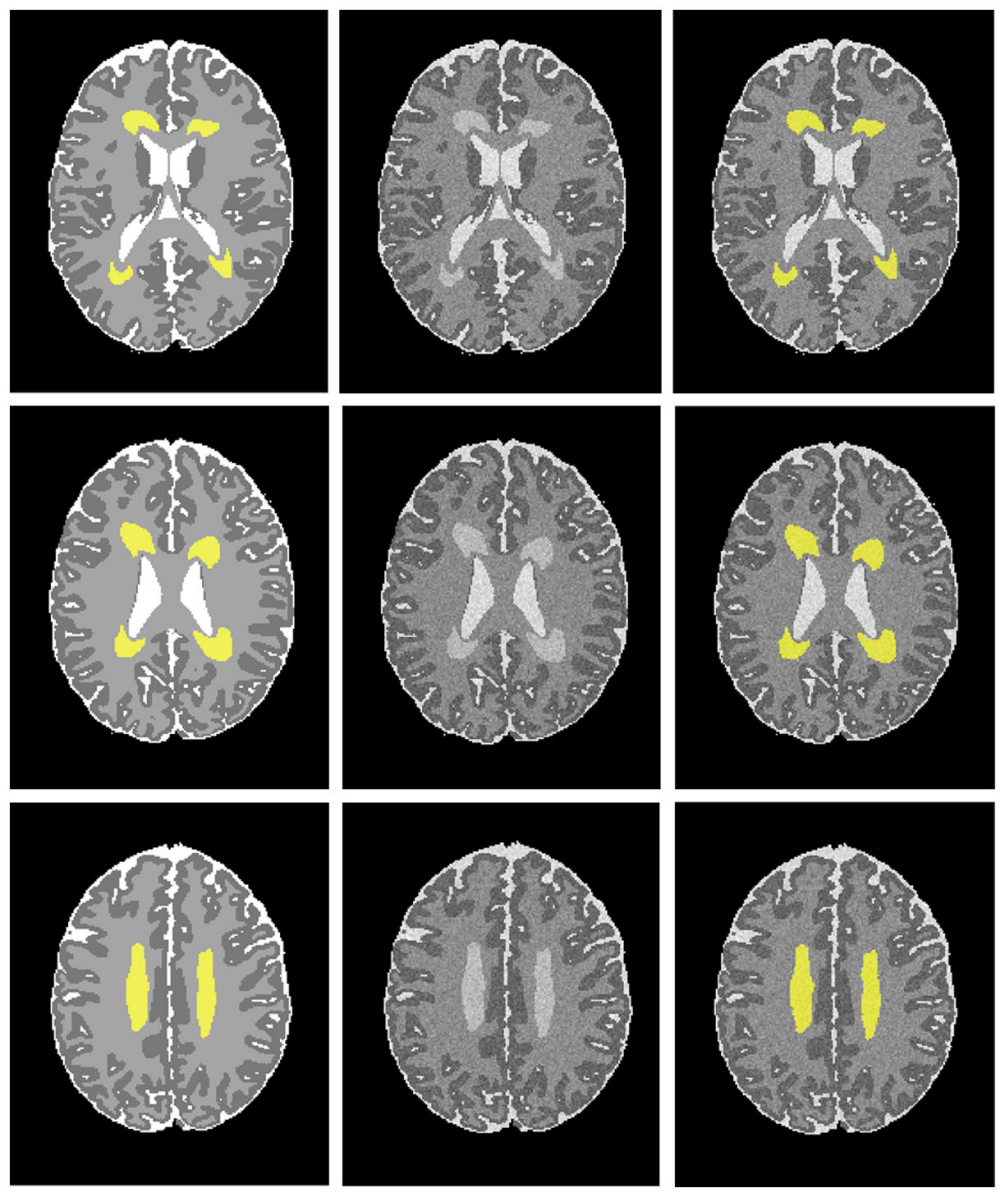
WMSA detection on simulated preterm T2-weighted brain images. Images at three mid-axial levels show, from left to right: noise-free images with manually drawn WMSA regions in yellow (ground truth); addition of Rician noise (SNR = 15) and INU (20% level); and WMSA detection by our proposed method marked in yellow.

Voxels with values greater than or equal to α SD above the mean of cerebral tissues (WM and GM) were defined to be WMSA, following the same strategy used in [18]. The cut-off threshold α was determined using computer simulations. Simulated preterm infant T2-weighted brain images with isotropic spatial resolution were constructed based on a normal anatomical model [32,33] combined with manually drawn synthetic WMSA regions (as shown in the first column in Figure 1.) by a neonatologist with more than a decade of experience in perinatal brain injury and MRI research. In contrast to adult brains, the intensity of WM is higher than that of GM. Heuristically, we set the mean values of CSF, WM, GM and WMSA to be 400, 260, 190 and 340 respectively. Different levels of Rician noise and multiplicative slowly-varying field of intensity non-uniformity (INU) with a complex shape and range of 0.9 to 1.1 (20% level) were imposed. Without loss of generality, we minimized the possible errors that spatial normalizations may cause by not varying brain sizes/shapes of each random Monte Carlo realization. WMSA detections using various α ranging from 1 to 1.8 were conducted and the one that facilitated maximum accuracy rates was considered to be optimal, irrespective of the level of noise, the optimal α was 1.4. 

Cerebral tissue segmentation is achieved by incorporating membership function from unified segmentation [34] with the anatomical information obtained by adapting very preterm infant probabilistic brain atlas to fit individual brain. More specifically, let *S* be a set of voxels/locations in a brain image and *x*_*i*_ (*i*∈*S*) be the corresponding intensities. The aim of the proposed algorithm is to segment images into different tissue regions (background, WM, GM, and CSF), say ω_*j*_, *j* = 1 to 4. We denote μ_*unified*_(ω_*j*_|x_*i*_) as membership function derived using the generative unified model provided in SPM (Wellcome Department of Cognitive Neurology, London, UK). Briefly, unified tissue segmentation requires the images to be normalized with tissue probability maps (refer to the section of very preterm infant probabilistic atlas creation). Spatial normalization is through non-linear deformation, which is modelled with a linear combination of non-linear 3D discrete cosine transforms basis functions. After normalization, these maps represent the prior probability of different tissue classes being found at each location in an image. Bayes rule can then be used to combine these priors with tissue type probabilities derived from voxel intensities to provide the posterior probability. In this unified segmentation model, intensity inhomogeneity correction was included in the mixture of Gaussian by extra parameters that account for smooth intensity variations. Partial volume artifact was controlled by assuming that the intensity distribution of each class may or may not be Gaussian (i.e. mixture of Gaussians). Typical numbers of Gaussians are three for grey matter and two for white matter, two for CSF and five for everything else.

**Figure 2:**
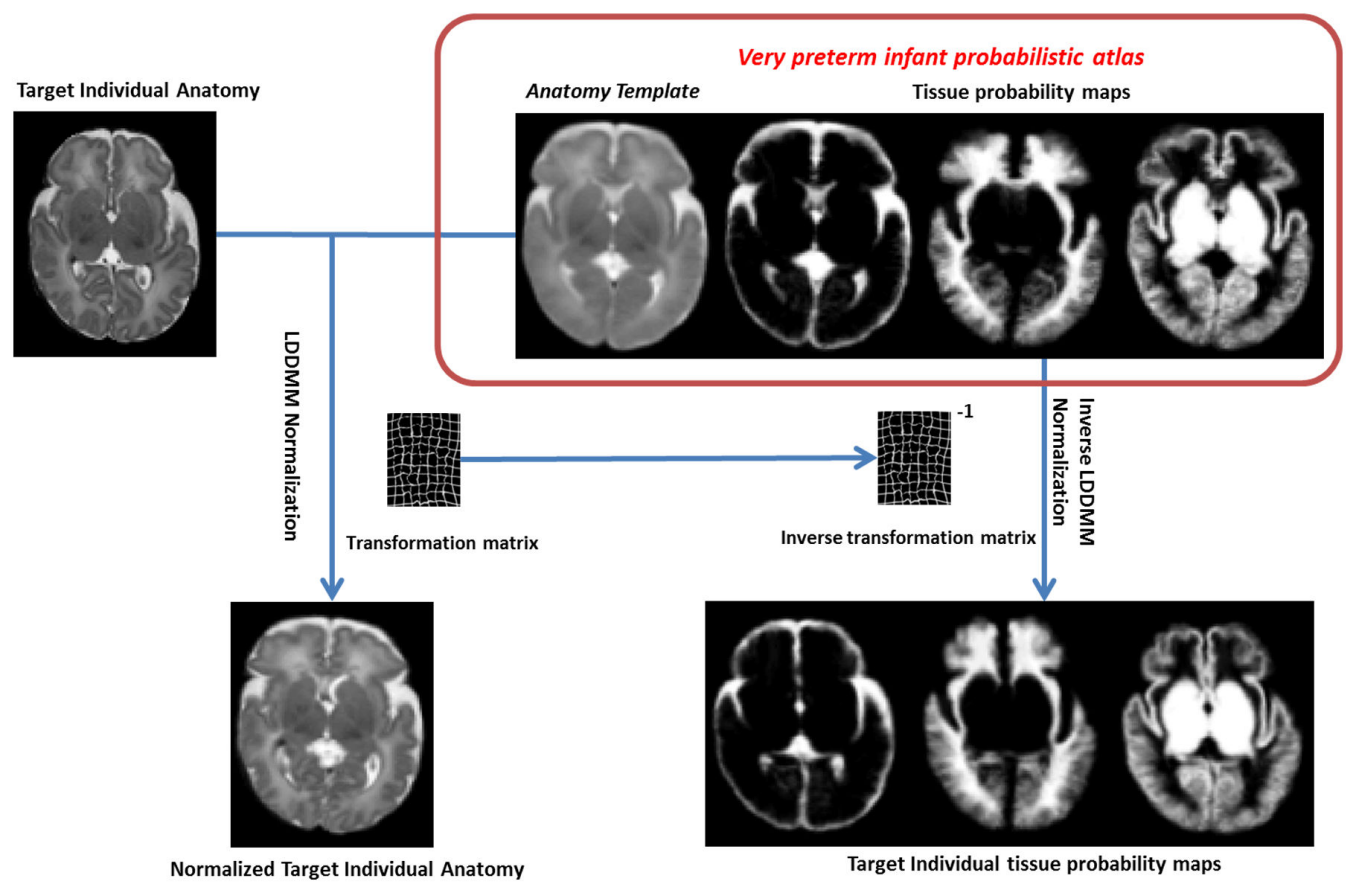
A flowchart of the generation of individual tissue probability maps. A target individual anatomy was first normalized to the reference space formed by the very preterm probabilistic atlas using LDDMM and the resultant transformation matrix was saved. The inverse transformation matric was applied to the tissue probability maps to create the desired target individual tissue probability maps.

We then propose to include an atlas-guided anatomy mislabelling correction into the model via individual tissue probability maps, μ_*atlas*_(ω_*j*_|x_*i*_), whose generation is illustrated in a flowchart shown in Figure 2. The final segmentation μ_ij_ is determined by a joint probabilistic membership function, 

$$\mu_{\text{ij}}=\frac{\mu_{unified}^{p}(\omega_{j}|x_{i})\cdot \mu_{atlas}^{q}(\omega_{j}|x_{i}))}{\sum_{c=1}^{4}\mu_{unified}^{p}(\omega_{c}|x_{i})\cdot \mu_{atlas}^{q}(\omega_{c}|x_{i})}$$

where *p* and *q* are two parameters controlling their respective contribution. 

**Figure 3:**
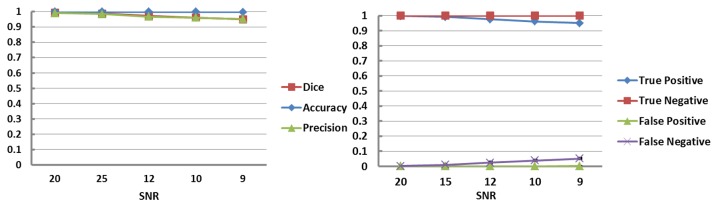
Comparison of automated WMSA detection on simulated infant MR images with ground truth. Quantitatively, automated detection showed very high Dice similarity index values (left) and low false detection rates (right) at each noise level with ground truth.

### Validation

By superimposing ground-truth and automated detections, four different pixel categories are generated: *a* (the number of correct detections that a voxel is negative), *b* (the number of incorrect detections that a voxel is positive), *c* (the number of incorrect detections that a voxel is negative), and *d* (the number of correct detections that a voxel is positive). Seven metrics are used: true positive rate = d / (*c* + *d*); true negative rate = *a* / (*a* + *b*); false positive rate = *b* / (*a* + *b*); false negative rate = *c* / (*c* + *d*); precision = d / (*b* + *d*); accuracy = (*a* + d) / (*a* + *b* + *c* + *d*) and Dice = (2 × precision × true positive) / (*precision* + *true positive*) [45].

### Language and cognitive developmental outcomes

All discharged ELBW infants were administered a standardized Bayley Scales of Infant and Toddler Development III (BSID-III) at 2 years of age. One infant died after discharge. Forty-seven of the remaining 49 infants (96%) returned for follow-up. However nine of these infants had to be excluded: seven exhibited behavioral problems and could not be tested for one or both Bayley scores; one infant had excessive motion artifacts; and one infant had severe encephalomalacia. Therefore, 38 ELBW infants were available for full analyses. The BSID-III language and cognitive subtests (on a scale of 50 to 150, with a mean of 100 and 150 indicating the most advanced development) were administered by a masked, certified examiner. All examiners are blinded to imaging findings during assessment. Bayley motor and behavioral scales were not administered to study infants. Standardized motor function was tested but was not included in our analyses because of the low incidence of cerebral palsy and resulting inadequate study power. 

### Statistical analysis

The relationships between continuous variables of objectively quantified WMSA volumes vs. BSID-III language and WMSA volumes vs. BSID-III cognitive scores were identified using both Pearson correlation and simple linear regression analyses. Assumptions of both analyses were tested and met. 

We use Pearson correlation coefficient *r* to measure the linear correlation between the two variables, giving a value between 1 and −1, where 1 is total positive correlation, 0 is no correlation, and −1 is negative correlation. In addition, we use linear regression to model the relationship between the two variables by fitting a linear equation. A linear regression line has an equation of the form *Y* = *a* + *bX*, where *b* is the slope of the line and *a* is the intercept. When *b* = 1 and *a* = 0, a perfect agreement can be said to exist between two variables. Coefficient of determination, denoted *R* ^2^ (0≤R^2^ ≤1) is used to describe how well the regression line fits the observed data. The larger the *R* ^2^, the better the fit.

In bivariate analyses, we tested the influence of sex, gestational age, and postmenstrual age at MRI scan, one at a time with WMSA volume and associated them with cognitive and language scores. Two-sided P values of <0.05 were considered to indicate statistical significance. No corrections were made for multiple comparisons [46].

## Results

### Simulations

The proposed method was first validated on simulated preterm infant brains with known ground-truth (manually drawn WMSA). Qualitatively, Figure 1 shows that the WMSA detection and quantification results have strong agreement with the ground truth. In addition, quantitative comparisons displayed in Figure 3 show very high similarity index values and low false detection rates at each signal-to-noise (SNR, which is the mean signal intensity divided by the standard deviation of the noise) level.

**Figure 4:**
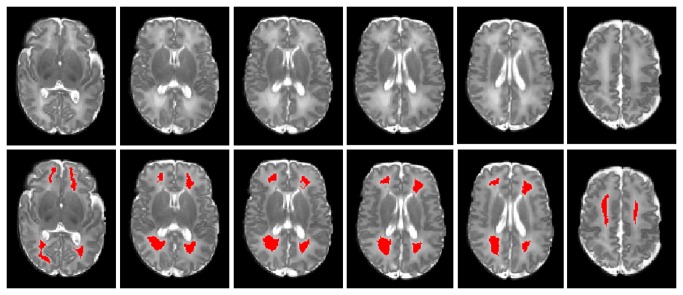
Automated WMSA detection at six different axial levels. Top row: T2-weighted images; bottom row: detected WMSA marked in red. The automated detections closely approximated the visually apparent signal abnormalities.

### In vivo very preterm data

Skull stripping was performed using the Brain Extraction Tool (BET) [35]. An experienced neonatologist visually inspected the detection results. Representative images from one ELBW infant with WMSA detection highlighted in red are presented in Figure 4. The automated WMSA detection closely approximated the visually apparent signal abnormalities.

**Figure 5:**
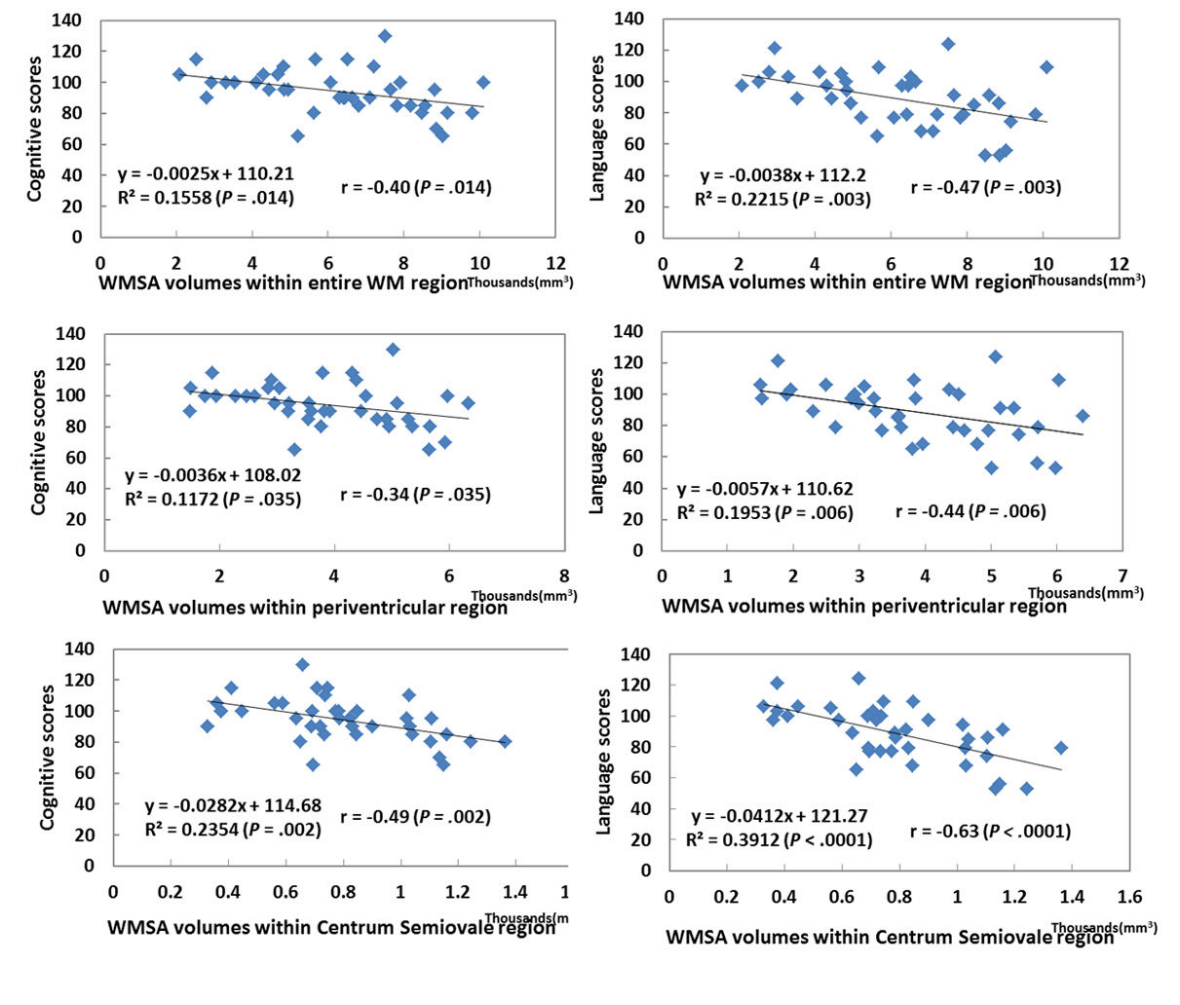
Linear regression and Pearson correlation analyses of automated quantified WMSA within different WM regions and Bayley III cognitive and language scores. *R* ^2^ denotes linear regression’s coefficient of determination and *r* denotes Pearson correlation coefficient. Larger WMSA volumes correspond with both lower cognitive and language scores. WMSA regional volume within centrum semiovale is a better predictor of Bayley scores than that within the periventricular WM regions.

### WMSA volume as a predictor of language and cognitive outcomes

WMSA volume was quantified by taking the product of total number of positive voxels in the detected binary mask by the volume of each voxel. The mean (SD) age at follow-up of our cohort was 23.7 (2.9) months (20.0 [2.8] months corrected age). The cohort BSID-III language scores had a mean of 94.3 and a standard deviation of 14.0; cognitive scores had a mean of 88.7 and a standard deviation of 17.4. Correlational statistical analyses showed that BSID-III language and cognitive scores were significantly (*P* value < 0.05) correlated with total WMSA volume as well as WMSA within the sub-regions of WM (Figure 5). WMSA at the level of the centrum semiovale exhibited the strongest linear correlation with Bayley scores, while WM in the periventricular crossroads exhibited the weakest correlation. These relationships modeled by linear regression are also plotted in Figure 5. In bivariate analyses, sex, gestational age, and postmenstrual age at MRI scan were not significant and exerted minimal influence on the beta coefficients.

## Discussions

Accurate and reproducible quantification of WMSA is critical for determining its developmental significance and potentially identifying high-risk preterm infants that may benefit from neuroprotective and early intervention therapies. In this paper, we present a fully automated atlas-guided method to detect and quantify WMSA on conventional T2-weighted imaging. Atlas-guided anatomic information was incorporated with unified segmentation and the relative importance of both components was controlled by two parameters. We assigned equal weights to both components, recognizing that different weighting would likely produce different results. Intuitively, if the knowledge carried by an atlas can be precisely forwarded to a target, μ_*atlas*_ should be weighted heavily; otherwise, one should consider weighing μ_*spm*_ heavily. Importantly, this approach likely contributed to our finding a significant association between WMSA volume and short-term standardized measures of language and cognitive development in ELBW infants.

Several studies have found that adult atlases do not form a suitable reference space for children and neonates, due to the considerable differences in size, geometrical proportions and brain tissue properties [36-39]. However, there are no studies on how well the atlases of full term neonates match very preterm infants, and vice versa. As compared to healthy term newborns, the brains of very preterm infants on TEA MRI frequently exhibit WMSA, delayed cortical maturation, and globally smaller structural and tissue volumes, even in the absence of signs of overt perinatal brain injury [40]. With more subjects being added to our current cohort study, this would be an interesting topic to explore. It is also worth noting that a dynamically time-varying atlas with dense duration coverage may more accurately reflect the fast rate of brain structure changes during the first few months of life [26,27,41-44]. 

Our studies were performed on unsedated infants who typically fall asleep after being fed and swaddled. The ability to now use conventional T2-weighted over multiple echo weighted imaging helps reduce scan time, resulting in less motion artifacts and need for sedation. Use of parallel imaging techniques, as used in our study, is also helping greatly in this regard. These innovations will facilitate larger multicenter imaging studies in very preterm infants and aid in definitively establishing the role of advanced brain MRI in this vulnerable population. Once more cases can be added to our preterm atlas, additional refinements such as localization of WMSA will be necessary to fully understand the developmental significance of WM hyperintensities in periventricular crossroads regions versus subcortical or centrum semiovale regions [12,13].

In our cohort of very preterm infants, we demonstrated automatically detected WMSA volume on TEA MRI to be a significant predictor of cognitive and language development at 2 years of age. Prior efforts to correlate WMSA with cognitive outcomes may have failed due to the low reliability of diagnosing WMSA qualitatively [9,14]. Our findings support the use of objective automated techniques to accurately quantify the lesion burden in perinatal-neonatal brain injury. Furthermore, lesion localization appears important in distinguishing developmentally normal from pathologic signal abnormalities, as observed in the periventricular crossroads and centrum semiovale regions, respectively. This also facilitates improved outcome prediction. 

The volume of WMSA in the centrum semiovale was the strongest predictor of Bayley scores, particularly language scores. This is consistent with findings from Krishnan et al. [6], who reported a significant correlation between apparent diffusion coefficient values in the centrum semiovale on TEA diffusion tensor imaging (DTI) and developmental quotient at 2 years corrected age. In a related study from this same cohort [47], we measured DTI microstructural measures in the centrum semiovale and found these to be independent predictors of cognitive/language scores at age 2. Our findings are also consistent with several other studies that found a relationship between WMSA and cognitive outcomes [4,5,7,48] but not all studies [8-11]. Similar to our study, both prior studies that used a quantitative measure of WMSA found a significant correlation with cognitive scores [6,48] but only a fraction of the studies that used a subjective diagnosis found a significant association. This suggests that measurement error may be playing a role in these variable outcomes, highlighting the need for objective quantitative diagnosis. Longer follow-up is also vital to assess if school-age outcome can be accurately predicted [49]. Due to the limited cohort size, we modeled the data using univariate regression only. For future larger studies, many other cofactors and clinical predictors can be incorporated to build a more complete and accurate multivariable model. Additional larger studies with school-age outcomes are needed to determine if WMSA in conjunction with other predictive factors could facilitate early infancy identification of individual infants at high risk for cognitive/language deficits and permit more intensive early intervention therapies. 

In summary, we constructed an age-specific very preterm infant probabilistic atlas. Guided with this atlas, we then proposed a fully automated method for WMSA detection and quantification using clinically available T2-weighted images. Computer simulations and validation using *in vivo* very preterm data showed very high detection accuracy. Correlational analyses demonstrated that WMSA volume at TEA predicted short-term language and cognitive developmental outcomes. Our work will facilitate population-based studies to more accurately characterize WMSA’s long-term sequelae. 
